# Supplementary material for: Cross-lineage protection by human antibodies binding the influenza B hemagglutinin
Source: Nat Commun. 2019 Jan 18;10:324. doi: 10.1038/s41467-018-08165-y (PMC6338745; doi:10.1038/s41467-018-08165-y)
Supplement: Supplementary file 1 — Supplementary Information [file 41467_2018_8165_MOESM1_ESM.pdf]

Supplementary Information for:

Cross-lineage protection by human antibodies binding the influenza B hemagglutinin

Liu et al.

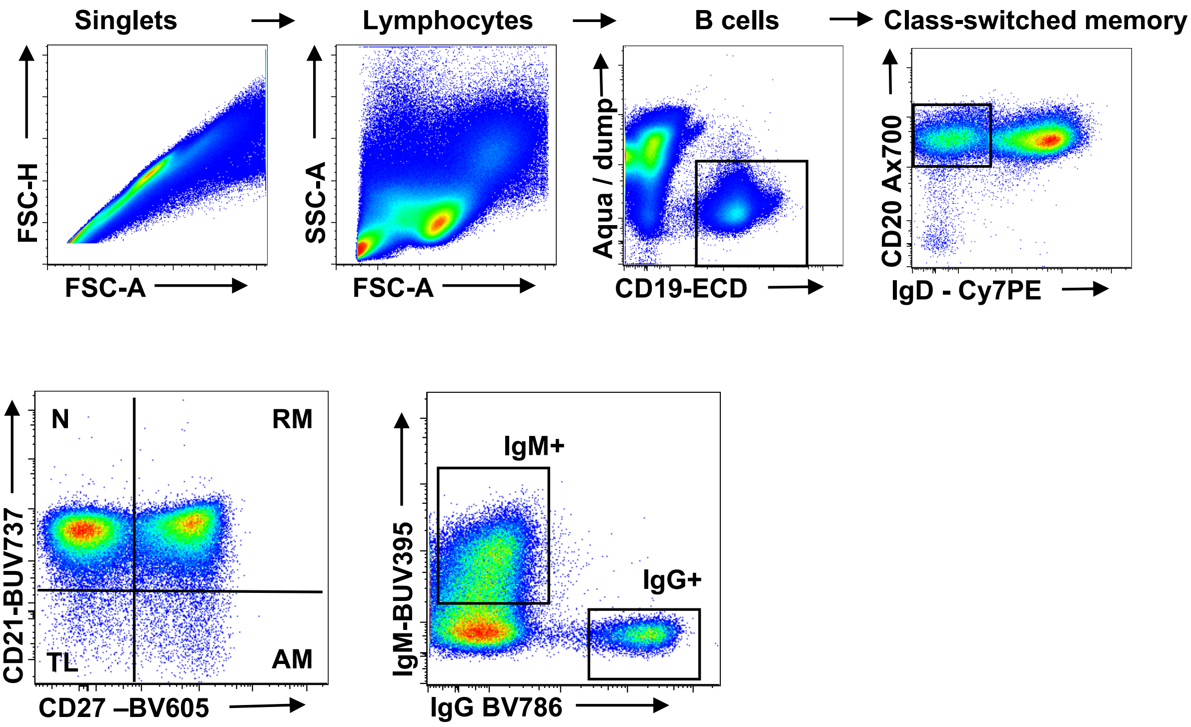

Supplementary Figure 1 - Representative flow cytometry gating

Single live CD19+ B lymphocytes were stained with IgD and CD20 to define class-switched B cells. Activation phenotype was assessed using surface markers CD21 and CD27. CD27- CD21+ naïve (N), CD27+ CD21+ resting memory (RM), CD27+ CD21- activated memory (AM) and CD27- CD21- tissue-like populations (TL) are denoted. Surface immunoglobulin expression was determined by co-staining for IgG and IgM subclasses. This gating was used for Figure 1 and 2.

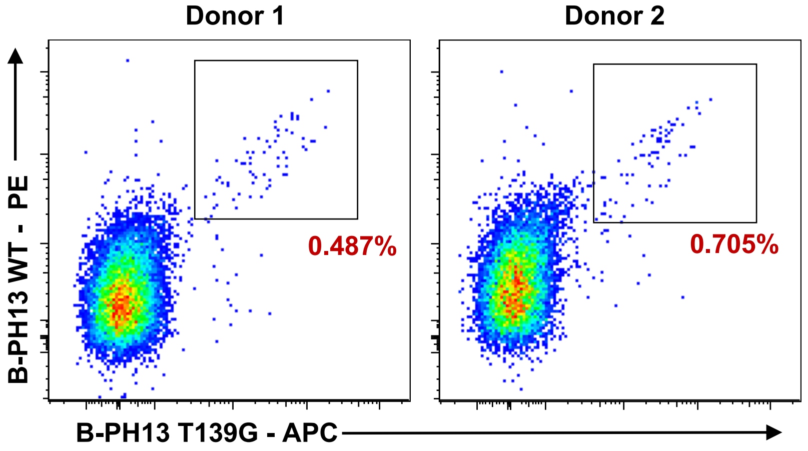

Supplementary Figure 2 - Co-staining memory B cells with wild-type (WT) and T139G recombinant HA probes from B/Phuket/3073/2013

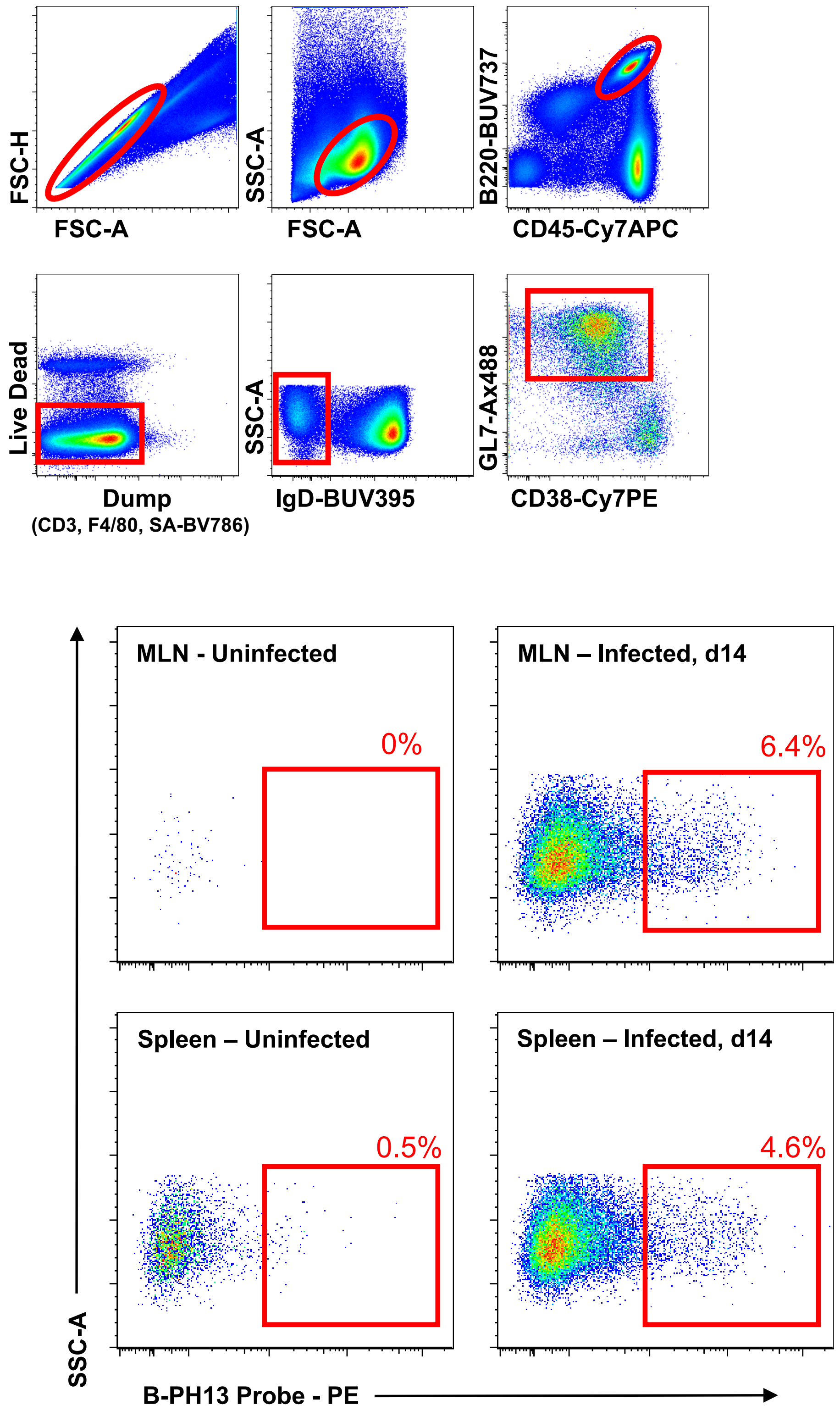

Supplementary Figure 3 – Ability of IBV HA probes to resolve HA-specific B cells was confirmed in infected mice. HA-specificity could be readily identified within germinal centre B cells isolated from the spleen or mediastinal lymph node of C57BL/6 mice infected intranasally with 10^4 $TCID_{50}$ B/Phuket/3073/2013.

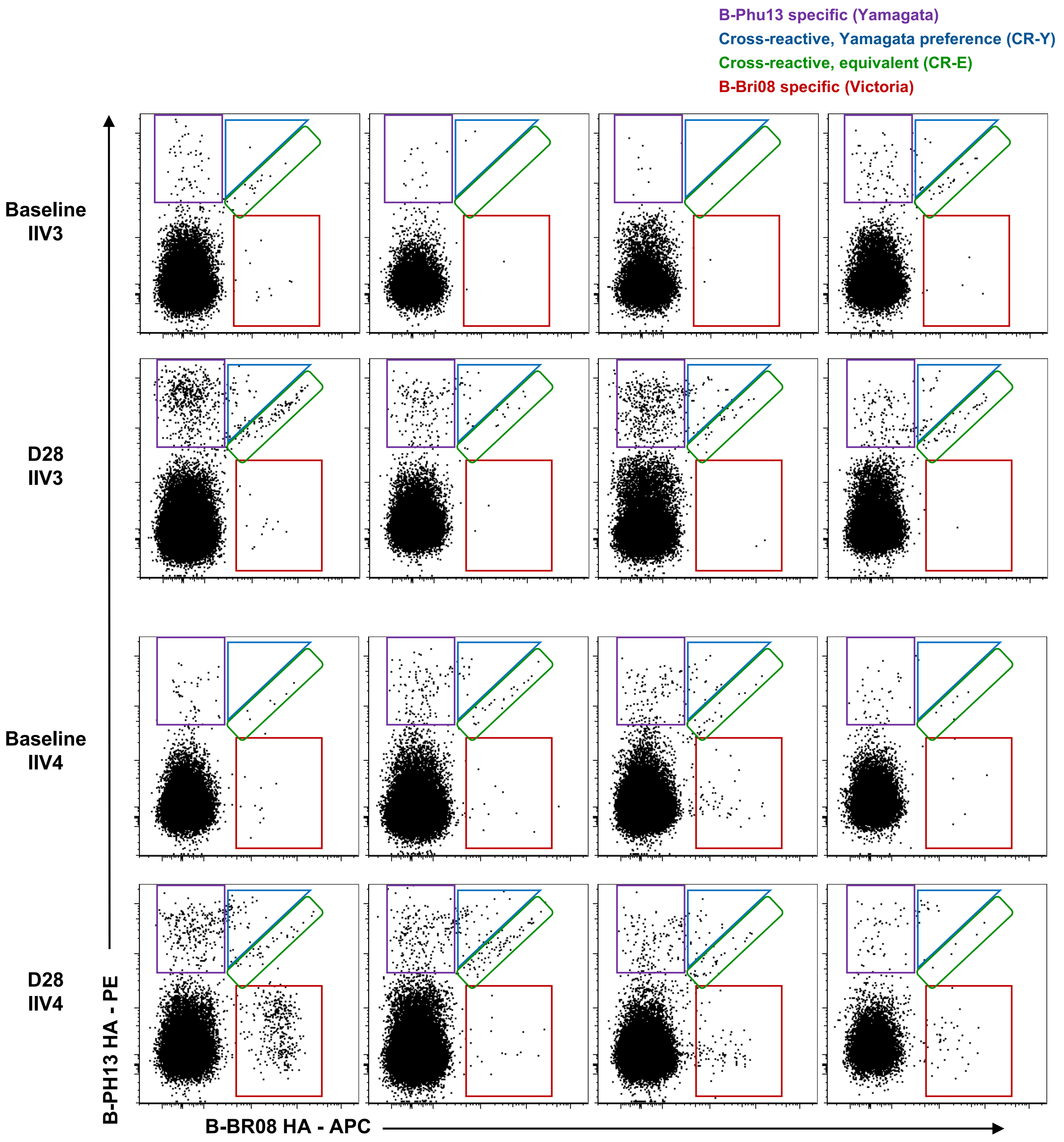

Supplementary Figure 4 - Changes in lineage-specific and cross-reactive B cell populations in a cross-section of 4 subjects receiving IIV3 and 4 subjects receiving IIV4

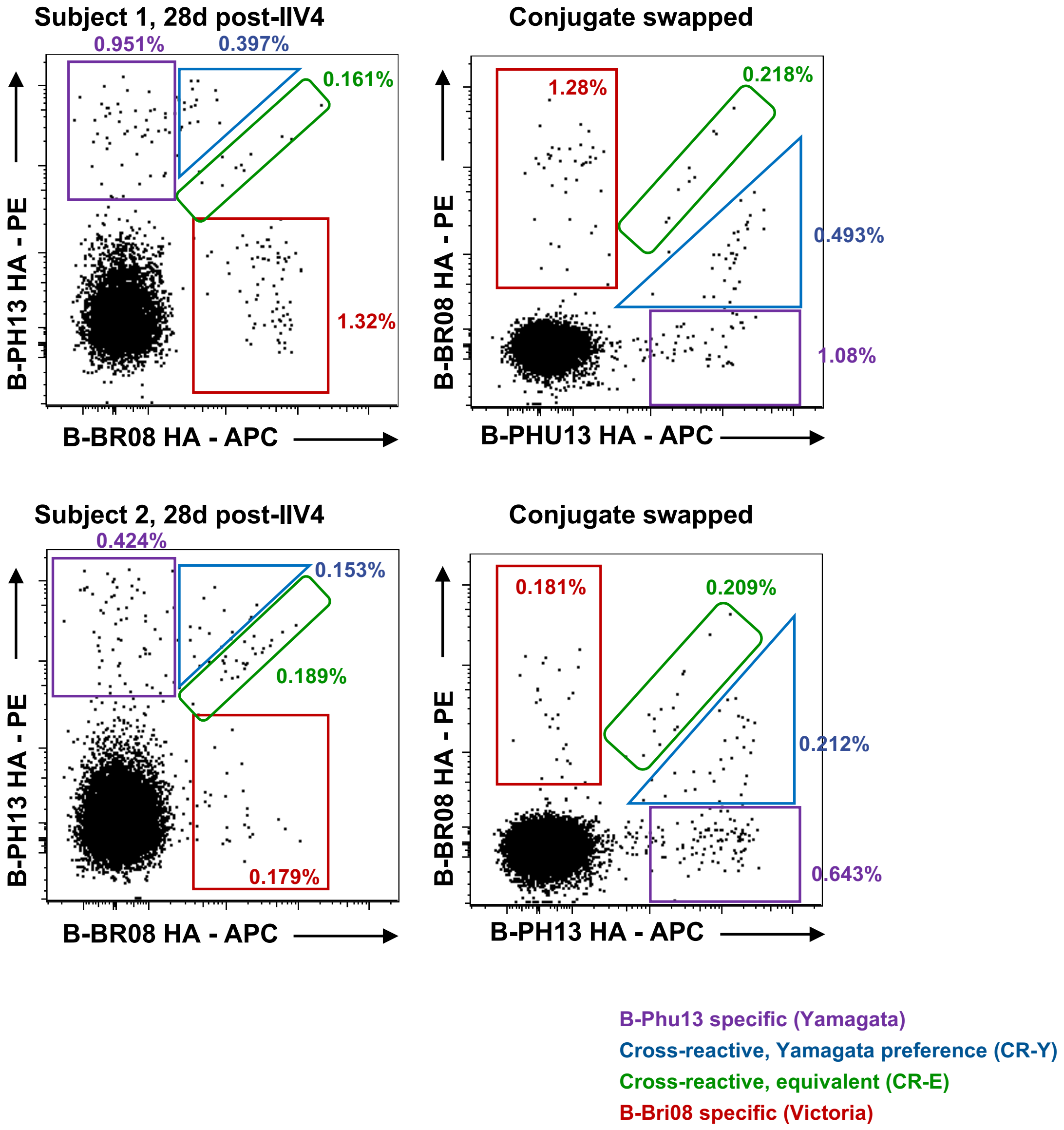

Supplementary Figure 5 - Distinct patterns of B-PH13 and B-BR08 cross-reactivity were confirmed using matched samples stained with swapped streptavidin conjugates.

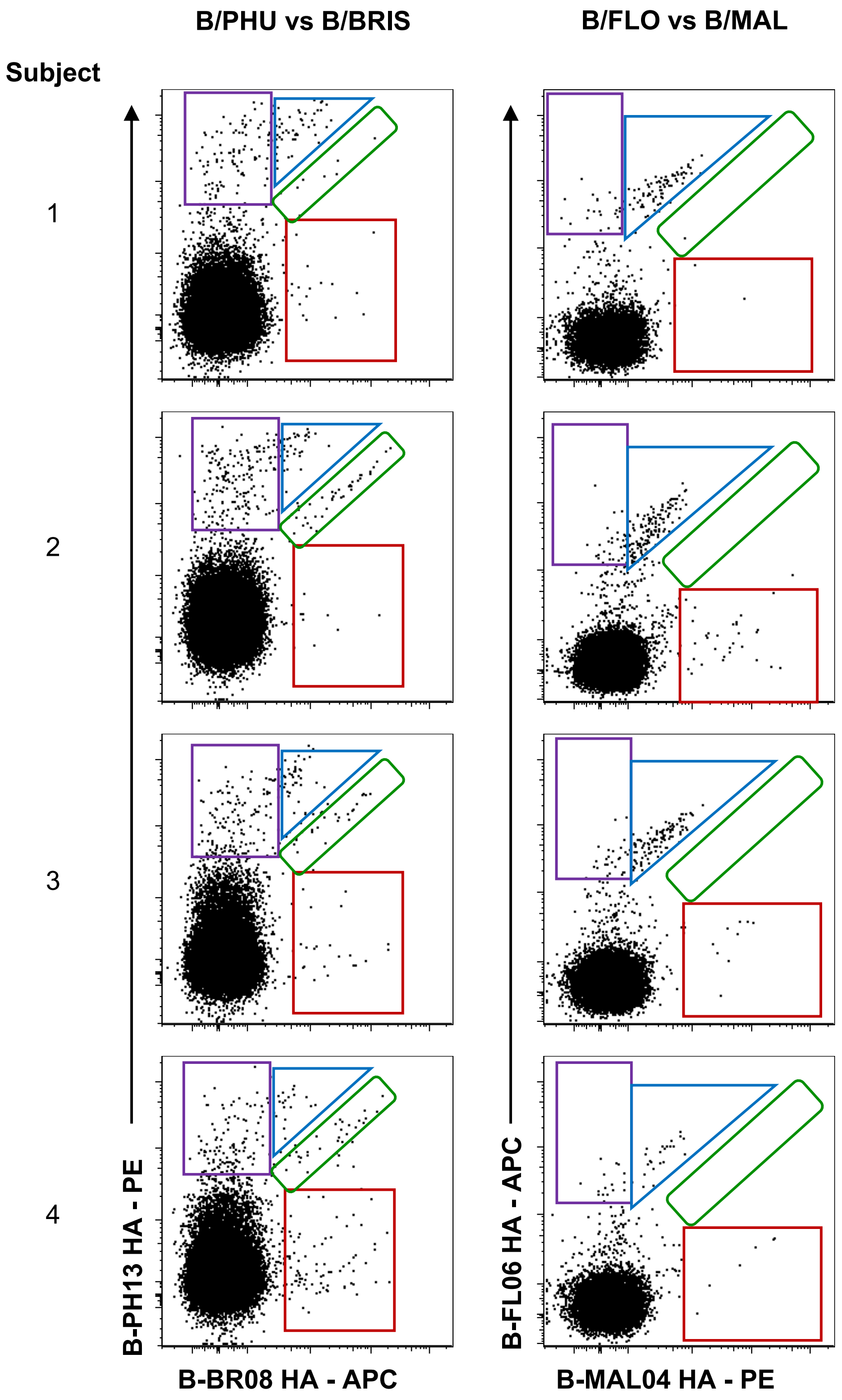

Supplementary Figure 6. B/Florida and B/Malaysia cross-reactive B cell populations in four representative subjects receiving IIV4

Cross-reactive staining patterns in cryopreserved PBMC samples from four subjects taken 4 weeks post-IIV4 immunisation were assessed using recombinant HA probes derived from B/Florida/4/2006 and B/Malaysia/2506/04. Shown in comparison to samples previously stained with B/BRIS and B/PHU probes.

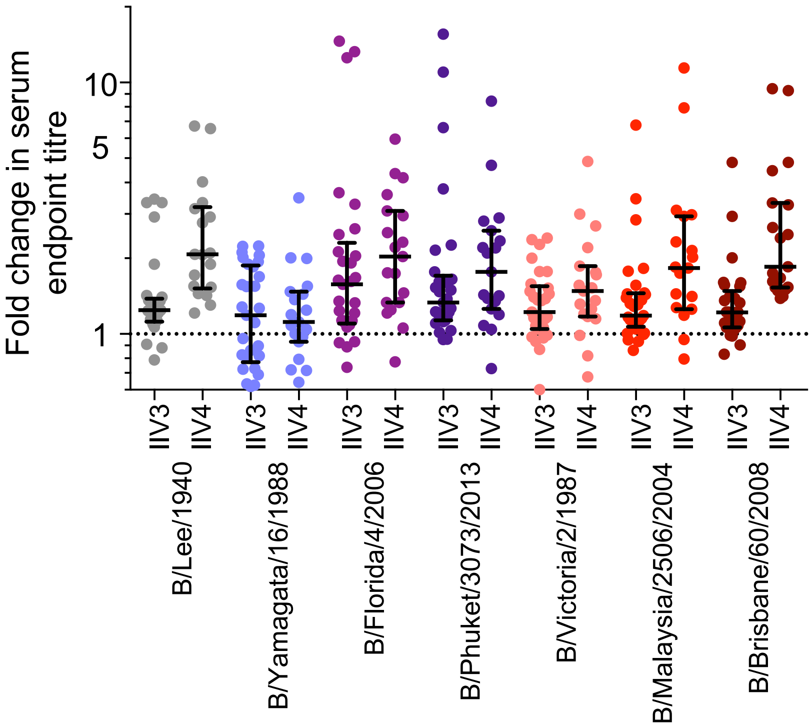

Supplementary Figure 7 – IBV HA-specific serum antibody responses following immunisation with seasonal influenza vaccines

The fold change in serum endpoint titres of antibody binding the indicated IBV strains following IIV3 and IIV4 immunisation was determined by ELISA. Median and IQR are indicated.

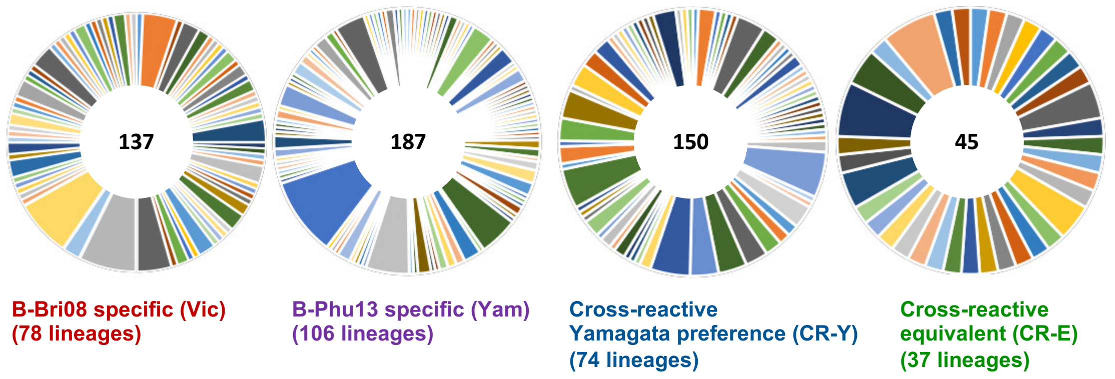

Supplementary Figure 8 – Clonal Distribution of recovered BCR transcripts

BCR sequences recovered from each sorted population were clustered into families based upon similarities in germline gene utilization, light chain pairings and the length and sequence of the CDR-H3. The clonal distribution of each population is shown using color coded lineages, the width proportional to the number of clones in each family and the total number of BCR recovered in each population indicated in the middle.

Sort Population	mAb	Clonal relatives	IGHV gene	IGHV mutation (%)	IGHD gene	IGHJ gene	CDR-H3	CDR-H3 length	IGKV/LV gene	IGKV/LV mutation (%)	IGKJ/LJ gene	CDR-L3 length
B-Bri08+	**K77-1C06**	3	4-39*07 F	7.22	6-19*01 F	4*02 F	CARVGQWLATNYFDYW	14	4-1*01 F	5.9	1*01 F	9
	**W85-1E03**	3	3-21*01 F, or 3-21*04 F	7.64	2-2*01 F	3*02 F	CARDIWGSNSRDDAFDIW	16	1-44*01 F	4.21	3*02 F	10
	**K77-2D11**	6	1-2*04 F	5.9	2-21*02 F	6*02 F	CARDDGYTTGADVW	12	3-25*03 F	2.87	3*02 F	10
	**W85-2C03**	6	3-13*04 F	8.77	2-21*01 F	4*02 F	CARLKPCGGTCYYLDYW	15	3-21*02 F	9.32	1*01 F	12
	**R95-1D08**	1	5-51*03 F	2.78	2-21*02 F	6*02 F	CARHETGDPGVYYHLDVW	16	3-15*01 F	5.11	4*01 F	9
	**K77-2E02**	3	4-59*08 F	4.91	2-2*01 F	6*02 F	CASAKRYCSSTSCYGLEYLFGMDVW	23	3-25*03 F	3.58	2*01 F, or 3*01 F or 3*02 F	11
	**W85-2D09**	2	1-46*01 F, or 1-46*03 F	5.21	2-21*02 F	4*02 F	CVRVGSCGGDCYFFDYW	15	3-21*03 F	1.43	3*02 F	11
	**R95-1D10**	3	1-46*01 F, or 1-46*03 F	3.82	2-21*02 F	4*02 F	CARTLSCGGDCYYFDFW	15	3-21*02 F	3.23	3*02 F	11
	**R95-1E06**	2	4-39*01 F	8.93	6-19*01 F	3*01 F, or 3*02 F	CARHQPWLVPSAFDFW	14	4-1*01 F	6.55	2*01 F, or 2*02 F	9
	**R95-1E12**	2	5-51*01 F	4.51	3-22*01 F	4*02 F	CARFGYDEIGQYYDFDYW	16	3-11*01 F	5.88	3*01 F	11
B-Phu13+	**W85-1B01**	2	3-43*02 F	5.56	5-18*01 F	3*02 F	CAKERPPPGHSFGLDAFDIW	18	3-21*02 F	3.23	2*01 F, or 3*01 F	10
	**R95-1E05**	7	1-69*02 F	11.11	3-22*01 F	6*03 F	CARDALYRPAYHDYYYYMDVW	19	3-10*01 F	11.11	3*02 F	11
	**K77-1G12**	2	3-20*01 F	7.64	6-6*01 F	6*02 F	CARARRASSSMDYYYYYGMDVW	20	1-33*01 F, or 1D-33*01 F	10.29	4*01 F	9
	**W85-1B01**	4	3-30*04 F, or 3-30-3*03 F	5.21	2-15*01 F	3*02 F	CARDRGLVVMDDTPDALDMW	18	1D-16*01 F	7.72	4*01 F	9
	**W85-1C08**	4	3-21*01 F	8.33	5-12*01 F	4*02 F	CTRDASGYDFDYW	11	2-14*01 F	4.86	1*01 F	10
	**K77-1H02**	4	3-43D*01 F	4.56	4-23*01 ORF	3*01 F, or 3*02 F	CARDSGRAVVNYVPEAFDFW	18	1D-16*01 F	6.57	4*01 F	9
	**W85-1C09**	9	3-30*04 F, or 3-30-3*03 F	5.56	2-2*01 F	4*02 F	CARDYCSTSSCYIDYW	14	2-14*01 F	1.39	1*01 F	10
	**W85-1D01**	3	4-59*08 F	5.61	2-21*01 F	6*03 F	CARLGAYCGGDCQPRNLYYSYYMDVW	24	2-28*01 F, or 2D-28*01 F	6.27	3*01 F	9
	**W85-2B04**	2	3-30*04 F, or 3-30-3*03 F	2.78	2-21*01 F	4*02 F	CARSHFYYDSGDYW	12	6-57*02 F	1.72	3*02 F	9
	**W85-3F06**	2	4-39*02 F	11.68	4-23*01 ORF	3*01 F	CARHSLLATVVTDAFDVW	16	3-11*01 F	8.96	4*01 F	11
Cross-reactive equivalent (CR-E)	**K77-1C02**	3	3-30*04 F, or 3-30-3*03 F	9.03	1-26*01 F	6*02 F	CARVVTENVYYSGMAVW	15	2-14*01 F	6.6	1*01 F	11
	**R95-1H09**	1	3-53*01 F	3.51	6-13*01 F	4*02 F	CARDGPPGDGSLDYW	13	3-21*02 F	1.08	2*01 F, or 3*01 F	10
	**K77-1G03**	1	3-64D*06 F	8.68	3-16*02 F	6*02 F	CVKGSYGDYDYYGMDVW	15	1-17*01 F	10.75	1*01 F	9
	**R95-1B11**	1	4-34*02 F	2.46	3-10*01 F	6*02 F	CARGPLRRIPMVRGVHDYAMDVW	21	3-20*01 F	2.13	5*01 F	10
	**K77-1G12**	2	3-30*02 F, or 3-30-5*02 F	7.99	3-16*01 F	4*02 F	CANAADEWGGGLQHDYW	15	2-14*01 F	8.33	3*02 F	10
	**R95-1C01**	1	1-69*01 F, or 1-69*05 F or 1-69D*01 F	6.6	2-21*02 F	6*02 F	CARDQPPWAETASSYSYGLDVW	20	2-30*01 F	6.64	4*01 F	10
	**R95-1D05**	1	3-30*01 F	9.03	3-9*01 F	6*02 F	CARDTYLYWYDVGHYHYYGMDVW	21	1-51*01 F	5.61	3*02 F	11
	**K77-2B06**	3	3-30*04 F, or 3-30-3*03 F	5.21	2-15*01 F	6*02 F	CAREKGEEEAGMDVW	13	4-1*01 F	3.12	3*01 F	9
	**K77-2D11**	1	1-18*01 F	6.6	3-10*01 F	6*02 F	CARDSYYGSGTYTPMNLYNYYYHMDVW	25	1-39*01 F, or 1D-39*01 F	8.09	2*01 F	10
	**R95-1E03**	1	1-2*04 F	4.17	6-25*01 F	4*02 F	CAKWQRGSGGGDYW	12	2-11*01 F	1.74	3*02 F	9
	**R95-1E05**	1	1-69*01 F, or 1-69D*01 F	8.33	5-24*01 ORF	3*02 F	CARDSPPWGDVNNNDAFDIW	18	2-30*01 F	5.94	5*01 F	10
	**K77-2H12**	1	3-7*03 F	5.9	4-17*01 F	4*02 F	CARDFSDFGDLYFDYW	14	2-11*01 F	2.43	3*02 F	10
	**R95-1E07**	1	3-30*02 F, or 3-30-5*02 F	11.11	3-3*02 F	4*02 F	CATEADDAVVGHDHDFW	15	2-14*01 F	5.21	3*02 F	11
	**R95-1F04**	1	3-7*01 F	3.82	3-3*01 F	6*02 F	CARDTFYEYWNRYYTYYYGMDVW	21	1-9*01 F	8.06	1*01 F	9
Cross-reactive Yamagata (CR-Y)	**W85-1A07**	4	3-48*03 F	6.25	4-17*01 F	3*02 F	CARAHMIYGDHVHLNAFDIW	18	2-14*01 F	4.17	3*02 F	10
	**R95-1H08**	5	3-30*03 F, or 3-30*18 F or 3-30-5*01 F	9.38	2-21*01 F	4*02 F	CARVFESYFFDYW	11	2-23*01 F, or 2-23*02 F or 2-23*03 F	6.94	3*02 F	10
	**K77-1H05**	4	3-30*04 F, or 3-30-3*03 F	7.64	1-1*01 F	4*02 F	CARTHLSYNFDYW	11	2-23*02 F	6.94	3*02 F	11
	**W85-3E10**	5	3-48*03 F	7.99	4-17*01 F	4*02 F	CARSGVVYGDSPAVFFDPW	17	3-15*01 F	7.27	2*01 F	10
	**R95-1H09**	4	3-33*01 F, or 3-33*06 F	6.6	2-2*02 F	6*02 F	CTRDPECTSSSCFSPTVSGMDVW	21	3-1*01 F	3.58	2*01 F, or 3*01 F	10
	**K77-2C08**	6	3-30*03 F, or 3-30*18 F or 3-30*19 F or 3-30-5*01 F	9.72	2-21*02 F	4*02 F	CVRVMDSYYFDYW	11	2-23*02 F	7.64	3*02 F	10
	**W85-1A07**	3	4-39*02 F	9.28	2-15*01 F	1*01 F	CARRTSGGNYYEYFHHW	15	2-28*01 F, or 2D-28*01 F	6.94	3*01 F	9
	**K77-2D09**	1	4-4*02 F	8.33	4-17*01 F	4*01 F, or 4*02 F	CAKSCKPGSCYYGDYLNFDSW	19	1-9*01 F	11.11	2*02 F	9
	**K77-2E07**	1	1-46*01 F, or 1-46*03 F	12.5	5-18*01 F	6*02 F	CARGRAEETWTQRWPPYSLDVW	20	3-20*01 F	4.96	3*01 F	10
	**R95-2A08**	3	3-66*01 F, or 3-66*04 F	9.12	2-21*02 F	3*02 F	CARDGGGWYVNDAFDIW	15	2-14*01 F	4.86	2*01 F, or 3*01 F	10
	**R95-2C02**	7	3-21*01 F	6.94	5-18*01 F	4*02 F	CARDEDAYGRVNYW	12	3-21*02 F	1.79	2*01 F, or 3*01 F	11
	**R95-2G10**	4	3-30*02 F, or 3-30-5*02 F	5.56	6-19*01 F	1*01 F	CAKAHNAAVAGLAEYFEHW	17	3-21*02 F	3.58	3*02 F	12

Supplementary Figure 9 - Genetic characteristics of IBV-specific human mAbs recovered from seasonal influenza vaccine recipients

A

```
B/Phuket/3073/2013 stem construct
MKAIIVLLMVVTSNADRICTGITSSNSPHVVKTATQGEVNVTGVIPLGSGLKLANGTKYRPQRETRGFFGAIAGFLEGGWEGMIA
GWHGYTSHGAHGVAVAADLKSTQEAINKITKNLNSLSELEGSGGSGTDLAELAVLLSNEGIINSEDEHLLALERKLKKMLGPSAV
DIGNGCFETKHKCNQTCLDRIAAGTFNAGEFSLPTFDSLNITGSGYIPEAPRDGQAYVRKDGEWVLLSTFLGSGLNDIFEAQKIE
WHEGHHHHHH*

B/Brisbane/60/2008 stem construct
MKAIIVLLMVVTSNADRICTGITSSNSPHVVKTATQGEVNVTGVIPLGSGLKLANGTKYRPQRETRGFFGAIAGFLEGGWEGMIA
GWHGYTSHGAHGVAVAADLKSTQEAINKITKNLNSLSELEGSGGSGTDLAELAVLLSNEGIINSEDEHLLALERKLKKMLGPSAV
EIGNGCFETKHKCNQTCLDRIAAGTFDAGEFSLPTFDSLNITGSGYIPEAPRDGQAYVRKDGEWVLLSTFLGSGLNDIFEAQKIE
WHEGHHHHHH*
```

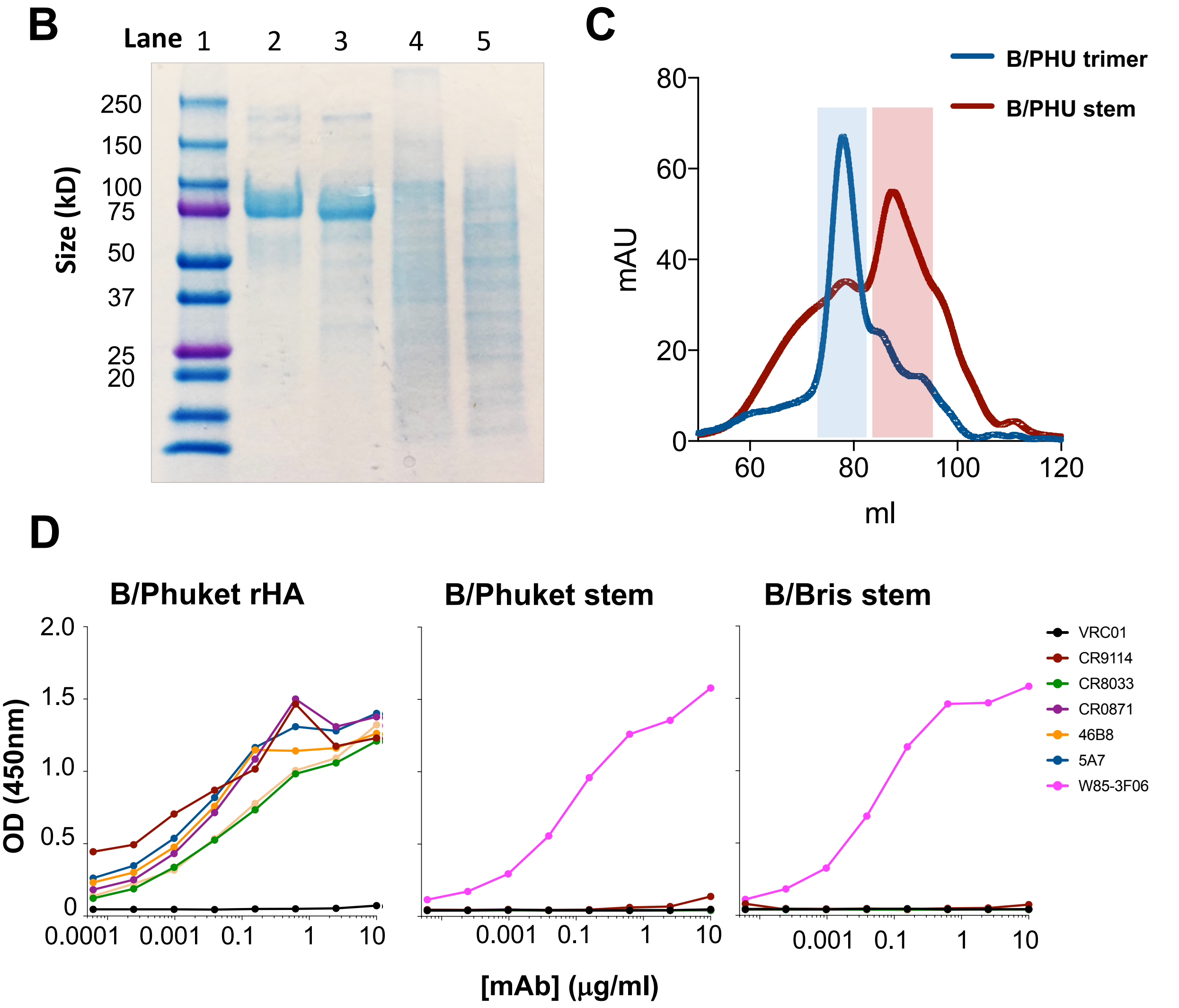

Supplementary Figure 10 – Design and expression of IBV HA stem proteins
(A) Stabilised IBV stem constructs encompassing relevant sections of the IBV HA ectodomain interspersed with linkers (purple) then C-terminally fused to the trimeric foldon of T4 fibritin (red), AviTag (green) and hexa-histidine affinity tag (blue). **(B)** SDS-PAGE of expressed recombinant IBV proteins. Lane 1 – marker, lane 2 – 5µg B/Brisbane/60/2008 HA trimer, lane 3 - 5µg B/Phuket/3073/2013 HA trimer, lane 4 - 5µg B/Brisbane/60/2008 HA stem, lane 5 - 5µg B/Phuket/3073/2013 HA stem. **(C)** Gel filtration trace of B/Phuket/3073/2013 HA trimer and B/Phuket/3073/2013 HA stem proteins. **(D)** Binding of known IBV-specific mAbs to stablised IBV stem proteins and a rHA control was examined by ELISA.

mAb	Virus	Mutant	IC50
K77-1G12	B/Phuket/3073/2013	WT	<0.05mg/ml
		G156E, T214I	>100mg/ml
W85-1B01	B/Phuket/3073/2013	WT	<0.05mg/ml
		G156R, D212N	>100mg/ml
R95-1D05	B/Phuket/3073/2013	WT	0.095mg/ml
		G156R	2.47mg/ml
K77-2E02	B/Brisbane/60/2008	WT	0.275mg/ml
		∆K177-D179, T214P	>100mg/ml
		T214P	>100mg/ml
CR8033	B/Phuket/3073/2013	WT	<0.05mg/ml
	B/Brisbane/60/2008	WT	44mg/ml
		∆K177-D179, T214P	>100mg/ml

Supplementary Figure 11 – Neutralisation activity of human mAbs against wild-type (WT) and escape mutant IBV

The inhibitory concentration of mAb sufficient the prevent infection of 50% of MDCK tissue culture wells (IC50) was determined for monoclonal wild-type and escape mutant viruses recovered after plaque purification.

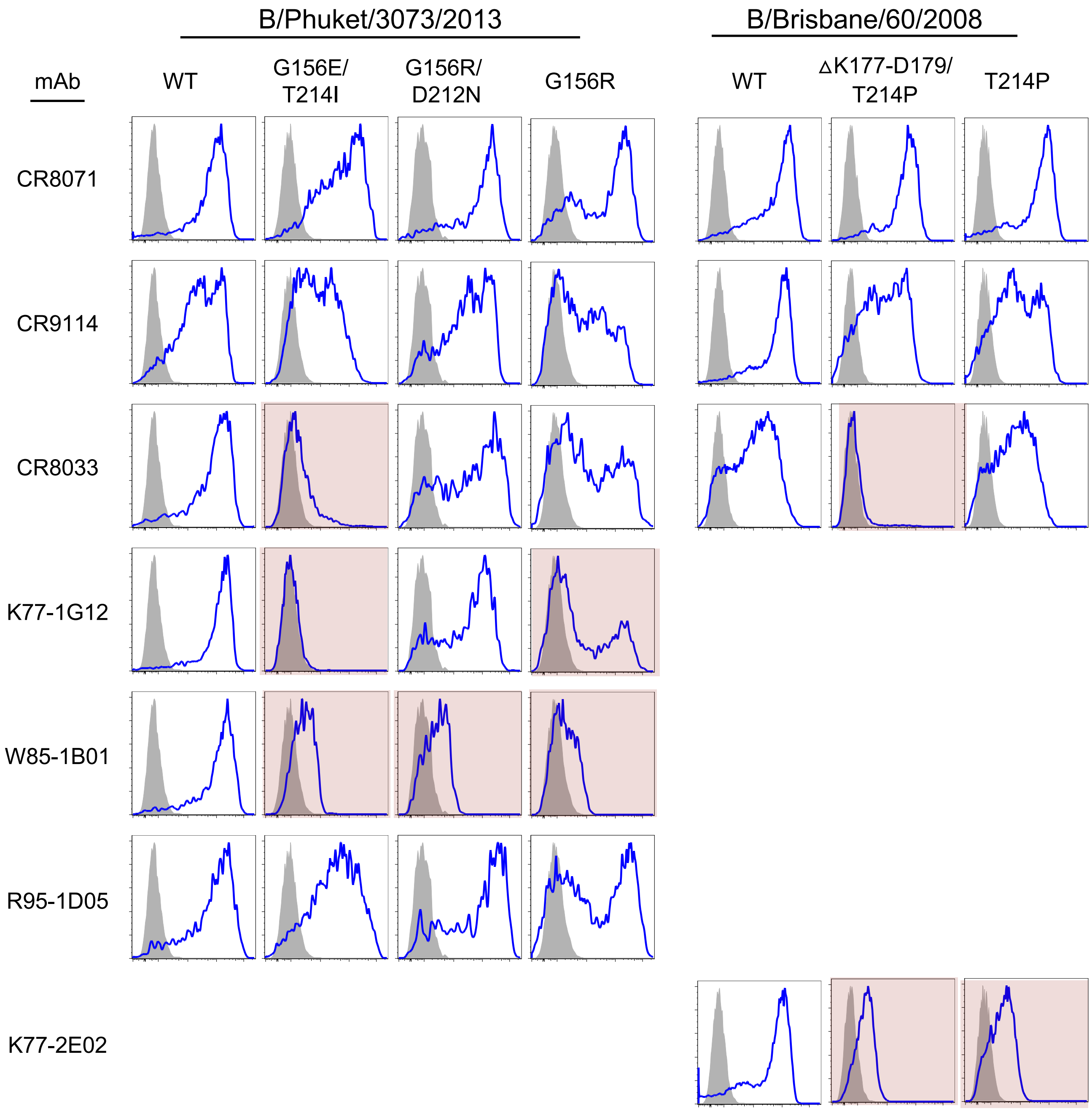

Supplementary Figure 12 – Binding of mAbs to surface HA following infection of MDCK cells

MDCK cells were infected *in vitro* with wild-type (WT) or viruses with the indicated escape mutations. The binding of human mAbs to HA on the cell surface was assessed by flow cytometry 18 hours post-infection. A major loss of binding relative to wild-type virus is indicated in red shading.

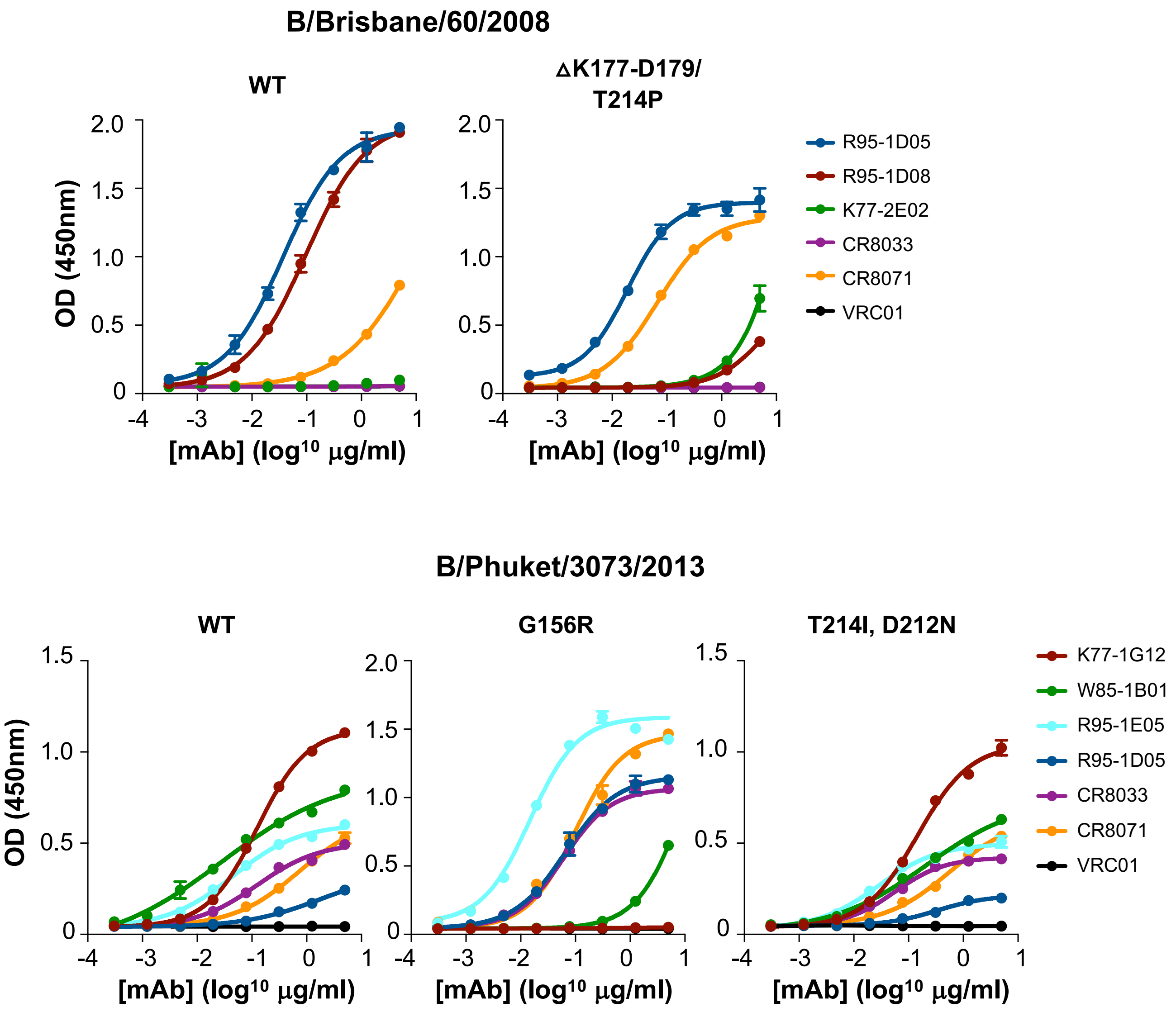

Supplementary Figure 13 - Binding of mAbs to wild-type and mutant HA by ELISA
Recombinant HA ectodomains were expressed using sequences from wild-type (WT) or viruses with the indicated escape mutations. The binding of human mAbs to HA was assessed by ELISA.

A

Cohort		2015 IIV3	2016 IIV4
**Age – mean (range)**		40.3 (22-55)	34.8 (21-53)
**Gender – #male (%)**		13 (43.3%)	10 (50%)
**Influenza vaccine history in prior 5 years (self-reported)**	*Unknown / not disclosed*	2 (6.7%)	3 (15%)
	0	2 (6.7%)	2 (10%)
	1	6 (20%)	5 (25%)
	2	4 (13.3%)	1 (5%)
	>3	16 (53.5%)	9 (45%)

B

Subject	Age	Gender	Influenza vaccine history in prior 5 years (self-reported)
**K77**	39	M	3
**W85**	31	F	1
**R95**	21	F	0

Supplementary Figure 14 – Summary of clinical trial participant information
(A) Summary of participant information from seasonal influenza vaccine immunisation trials in 2015 and 2016. **(B)** Details of participants used for B cell sorting and recovery of monoclonal antibodies.

B/Brisbane/60/2008
MKAIIVLLMVVTSNADRICTGITSSNSPHVVKTATQGEVNVTGVIPLTTTPTKSHFANLKGTETRGKLCPKCLNCTDLDVALGRP
KCTGKIPSARVSILHEVRPVTSGCFPIMHDRTKIRQLPNLLRGYEHIRLSTHNVINAENAPGGPYKIGTSGSCPNITNGNGFFAT
MAWAVPKNDKNKTATNPLTIEVPYICTEGEDQITVWGFHSDDETQMAKLYGDSKPQKFTSSANGVTTHYVSQIGGFPNQTEDGGL
PQSGRIVVDYMVQKSGKTGTITYQRGILLPQKVWCASGRSKVIKGSLPLIGEADCLHEKYGGLNKSKPYYTGEHAKAIGNCPIWV
KTPLKLANGTKYRPPAKLLKERGFFGAIAGFLEGGWEGMIAGWHGYTSHGAHGVAVAADLKSTQEAINKITKNLNSLSELEVKNL
QRLSGAMDELHNEILELDEKVDDLRADTISSQIELAVLLSNEGIINSEDEHLLALERKLKKMLGPSAVEIGNGCFETKHKCNQTC
LDRIAAGTFDAGEFSLPTFDSLNITGSGYIPEAPRDGQAYVRKDGEWVLLSTFLGSGLNDIFEAQKIEWHEGHHHHHH*

B/Phuket/3073/2013
MKAIIVLLMVVTSNADRICTGITSSNSPHVVKTATQGEVNVTGVIPLTTTPTKSYFANLKGTRTRGKLCPDCLNCTDLDVALGRP
MCVGTTPSAKASILHEVRPVTSGCFPIMHDRTKIRQLPNLLRGYEKIRLSTQNVIDAEKAPGGPYRLGTSGSCPNATSKIGFFAT
MAWAVPKDNYKNATNPLTVEVPYICTEGEDQITVWGFHSDNKTQMKSLYGDSNPQKFTSSANGVTTHYVSQIGDFPDQTEDGGLP
QSGRIVVDYMMQKPGKTGTIVYQRGVLLPQKVWCASGRSKVIKGSLPLIGEADCLHEEYGGLNKSKPYYTGKHAKAIGNCPIWVK
TPLKLANGTKYRPPAKLLKERGFFGAIAGFLEGGWEGMIAGWHGYTSHGAHGVAVAADLKSTQEAINKITKNLNSLSELEVKNLQ
RLSGAMDELHNEILELDEKVDDLRADTISSQIELAVLLSNEGIINSEDEHLLALERKLKKMLGPSAVDIGNGCFETKHKCNQTCL
DRIAAGTFNAGEFSLPTFDSLNITGSGYIPEAPRDGQAYVRKDGEWVLLSTFLGSGLNDIFEAQKIEWHEGHHHHHH*

B/Malaysia/2506/04
MKAIIVLLMVVTSNADRICTGITSSNSPHVVKTATQGEVNVTGVIPLTTTPTKSHFANLKGTETRGKLCPKCLNCTDLDVALGRP
KCTGNIPSARVSILHEVRPVTSGCFPIMHDRTKIRQLPNLLRGYEHIRLSTHNVINAENAPGGSYKIGTSGSCPNVTNGNGFFAT
MAWAVPKNDNNKTATNSLTIEVPYICTEGEDQITVWGFHSDNEAQMAKLYGDSKPQKFTSSANGVTTHYVSQIGGFPNQTEDGGL
PQSGRIVVDYMVQKSGKTGTITYQRGILLPQKVWCASGRSKVIKGSLPLIGEADCLHEKYGGLNKSKPYYTGEHAKAIGNCPIWV
KTPLKLANGTKYRPPAKLLKERGFFGAIAGFLEGGWEGMIAGWHGYTSHGAHGVAVAADLKSTQEAINKITKNLNSLSELEVKNL
QRLSGAMDELHNEILELDEKVDDLRADTISSQIELAVLLSNEGIINSEDEHLLALERKLKKMLGPSAVEIGNGCFETKHKCNQTC
LDRIAAGTFDAGEFSLPTFDSLNITGSGYIPEAPRDGQAYVRKDGEWVLLSTFLGSGLNDIFEAQKIEWHEGHHHHHH*

B/Florida/60/2008
MKAIIVLLMVVTSNADRICTGITSSNSPHVVKTATQGEVNVTGVIPLTTTPTKSYFANLKGTRTRGKLCPDCLNCTDLDVALGRP
MCVGTTPSAKASILHEVKPVTSGCFPIMHDRTKIRQLPNLLRGYENIRLSTQNVIDAEKAPGGPYRLGTSGSCPNATSKSGFFAT
MAWAVPKDNNKNATNPLTVEVPYICTEGEDQITVWGFHSDDKTQMKNLYGDSNPQKFTSSANGVTTHYVSQIGSFPDQTEDGGLP
QSGRIVVDYMMQKPGKTGTIVYQRGVLLPQKVWCASGRSKVIKGSLPLIGEADCLHEKYGGLNKSKPYYTGEHAKAIGNCPIWVK
TPLKLANGTKYRPPAKLLKERGFFGAIAGFLEGGWEGMIAGWHGYTSHGAHGVAVAADLKSTQEAINKITKNLNSLSELEVKNLQ
RLSGAMDELHNEILELDEKVDDLRADTISSQIELAVLLSNEGIINSEDEHLLALERKLKKMLGPSAVEIGNGCFETKHKCNQTCL
DRIAAGTFNAGEFSLPTFDSLNITGSGYIPEAPRDGQAYVRKDGEWVLLSTFLGSGLNDIFEAQKIEWHEGHHHHHH*

B/Victoria/2/1987
MKAIIVLLMVVTSNADRICTGITSSNSPHVVKTATQGEVNVTGVIPLTTTPTKSHFANLKGTKTRGKLCPKCLNCTDLDVALARP
KCMGTIPSAKASILHEVKPVTSGCFPIMHDRTKIRQLPNLLRGYEHIRLSTHNVINAETAPGGPYKVGTSGSCPNVTNGNGFFAT
MAWAVPKNDNNKTATNPLTVEVPYICTEGEDQITVWGFHSDSETQMVKLYGDSKPQKFTSSANGVTTHYVSQIGGFPNQAEDGGL
PQSGRIVVDYMVQKSGKTGTITYQRGILLPQKVWCASGRSKVIKGSLPLIGEADCLHEKYGGLNKSKPYYTGEHAKAIGNCPIWV
KTPLKLANGTKYRPPAKLLKERGFFGAIAGFLEGGWEGMIAGWHGYTSHGAHGVAVAADLKSTQEAINKITKNLNSLSELEVKNL
QRLSGAMDELHNEILELDEKVDDLRADTISSQIELAVLLSNEGIINSEDEHLLALERKLKKMLGPSAVEIGNGCFETKHKCNQTC
LDRIAAGTFNAGEFSLPTFDSLNITGSGYIPEAPRDGQAYVRKDGEWVLLSTFLGSGLNDIFEAQKIEWHEGHHHHHH*

B/Yamagata/16/1988
MKAIIVLLMVVTSNADRICTGITSSNSPHVVKTATQGEVNVTGVIPLTTTPTKSHFANLKGTKTRGKLCPNCLNCTDLDVALGRP
MCMGTIPSAKASILHEVRPVTSGCFPIMHDRTKIRQLPNLLRGYENIRLSTHNVINAERAPGGPYRLGTSGSCPNVTSRNGFFAT
MAWAVPRDNKTATNPLTVEVPYICTKGEDQITVWGFHSDDKTQMKNLYGDSNPQKFTSSANGVTTHYVSQIGDFPNQTEDGGLPQ
SGRIVVDYMVQKPGKTGTIVYQRGVLLPQKVWCASGRSKVIKGSLPLIGEADCLHEKYGGLNKSKPYYTGEHAKAIGNCPIWVKT
PLKLANGTKYRPPAKLLKERGFFGAIAGFLEGGWEGMIAGWHGYTSHGAHGVAVAADLKSTQEAINKITKNLNSLSELEVKNLQR
LSGAMDELHNEILELDEKVDDLRADTISSQIELAVLLSNEGIINSEDEHLLALERKLKKMLGPSAVDIGNGCFETKHKCNQTCLD
RIAAGTFNAGEFSLPTFDSLNITGSGYIPEAPRDGQAYVRKDGEWVLLSTFLGSGLNDIFEAQKIEWHEGHHHHHH*

B/Lee/1940
MKAIIVLLMVVTSNADRICTGITSSNSPHVVKTATQGEVNVTGVIPLTTTPTRSHFANLKGTQTRGKLCPNCFNCTDLDVALGRP
KCMGNIPSAKVSILHEVKPVTSGCFPIMHDRTKIRQLPNLLRGYENIRLSTSNVINAETAPGGPYKVGTSGSCPNVANRNGFFNT
MAWVIPKDNNKTAINPVTVEVPYICSEGEDQITVWGFHSDDKTQMERLYGDSNPQKFTSSANGVTTHYVSQIGGFPNQTEDEGLK
QSGRIVVDYMVQKPGKTGTIVYQRGILLPQKVWCASGRSKVIKGSLPLIGEADCLHEKYGGLNKSKPYYTGEHAKAIGNCPIWVK
TPLKLANGTKYRPPAKLLKERGFFGAIAGFLEGGWEGMIAGWHGYTSHGAHGVAVAADLKSTQEAINKITKNLNSLSELEVKNLQ
RLSGAMNGLHDEILELDEKVDDLRADTISSQIELAVLLSNEGIINSEDEHLLALERKLKKMLGPSAVEIGNGCFETKHKCNQTCL
DRIAAGTFNAGDFSLPTFDSLNITGSGYIPEAPRDGQAYVRKDGEWVLLSTFLGSGLNDIFEAQKIEWHEGHHHHHH*

Supplementary Figure 15 - Recombinant HA probe sequences
The indicated IBV HA ectodomain was C-terminally fused to the trimeric foldon of T4 fibritin (red), AviTag (green) and hexa-histidine affinity tag (blue).

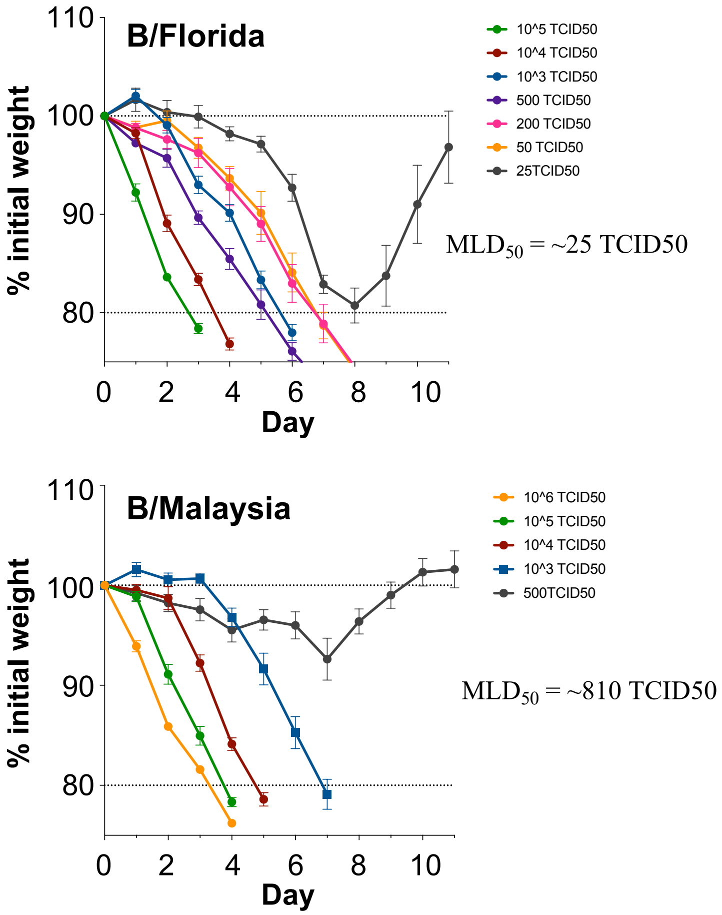

Supplementary Figure 16 - Titration of mouse challenge stocks
Weight loss in mice (N=5 per group) receiving increasing intranasal doses of B/Florida/4/2006 or B/Malaysia/2506/2004 challenge stocks. Data are mean and SEM.
