# Supplementary material for: Cross-lineage protection by human antibodies binding the influenza B hemagglutinin
Source: Nat Commun. 2019 Jan 18;10:324. doi: 10.1038/s41467-018-08165-y (PMC6338745; doi:10.1038/s41467-018-08165-y)
Supplement: Supplementary file 3 — Description of Additional Supplementary Files [file 41467_2018_8165_MOESM3_ESM.docx]

**Description of Additional Supplementary Files**

**File Name:** Supplementary data 1

**Description:** Recovered heavy and light chain immunoglobulin sequences from single sorted

IBV-specific memory B cells.
